# The role of polygenic risk and susceptibility genes in breast cancer over the course of life

**DOI:** 10.1038/s41467-020-19966-5

**Published:** 2020-12-14

**Authors:** Nina Mars, Elisabeth Widén, Sini Kerminen, Tuomo Meretoja, Matti Pirinen, Pietro della Briotta Parolo, Priit Palta, FinnGen, Aki Havulinna, Amanda Elliott, Anastasia Shcherban, Andrea Ganna, Anu Jalanko, Arto Lehisto, Elina Kilpeläinen, Georg Brein, Ghazal Awaisa, Hannele Laivuori, Henrike Heyne, Jarmo Harju, Jiwoo Lee, Juha Karjalainen, Jukka Koskela, Kalle Pärn, Kati Donner, Kristin Tsuo, Manuel González Jiménez, Mari Kaunisto, Mari E. Niemi, Mary Pat Reeve, Mervi Aavikko, Mitja Kurki, Oluwaseun A. Dada, Pietro della Briotta Parolo, Risto Kajanne, Sina Rüeger, Susanna Lemmelä, Taru Tukiainen, Tiinamaija Tuomi, Timo P. Sipilä, Tuomo Kiiskinen, Vincent Llorens, Adam Ziemann, Anne Lehtonen, Apinya Lertratanakul, Bob Georgantas, Bridget Riley-Gillis, Danjuma Quarless, Fedik Rahimov, Howard Jacob, Jeffrey Waring, Justin Wade Davis, Nizar Smaoui, Relja Popovic, Sahar Esmaeeli, Athena Matakidou, Ben Challis, David Close, Eleonor Wigmore, Slavé Petrovski, Chia-Yen Chen, Ellen Tsai, Heiko Runz, Jimmy Liu, Paola Bronson, Sally John, Sanni Lahdenperä, Stephanie Loomis, Susan Eaton, Yunfeng Huang, Erika Kvikstad, Minal Caliskan, Samir Wadhawan, Elmutaz Shaikho Elhaj Mohammed, Janet van Adelsberg, Joseph Maranville, Marla Hochfeld, Robert Plenge, Shameek Biswas, Steven Greenberg, Andrew Peterson, David Choy, Diana Chang, Edmond Teng, Erich Strauss, Geoff Kerchner, Hao Chen, Hubert Chen, Jennifer Schutzman, John Michon, Julie Hunkapiller, Mark McCarthy, Natalie Bowers, Sarah Pendergrass, Tushar Bhangale, David Pulford, Dawn Waterworth, Diptee Kulkarni, Fanli Xu, Jo Betts, Jorge Esparza Gordillo, Joshua Hoffman, Karen S. King, Meg Ehm, Soumitra Ghosh, Patrick Loerch, Wilco Fleuren, Beryl Cummings, Matt Brauer, Robert Graham, Tim Behrens, Andrey Loboda, Anna Podgornaia, Aparna Chhibber, Audrey Chu, Caroline Fox, Dorothee Diogo, Emily Holzinger, John Eicher, Padhraig Gormley, Vinay Mehta, Anders Malarstig, Åsa Hedman, Catherine Marshall, Christopher Whelan, Heli Lehtonen, Jaakko Parkkinen, Kari Linden, Kirsi Kalpala, Melissa Miller, Nan Bing, Stefan McDonough, Xing Chen, Xinli Hu, Ying Wu, Anthony Muslin, Clarence Wang, Clement Chatelain, Deepak Rajpal, Dongyu Liu, Ethan Xu, Franck Auge, Katherine Call, Kathy Klinger, Marika Crohns, Matthias Gossel, Tai-he Xia, Minna Hendolin, Outi Tuovila, Outi Tuovila, Raimo Pakkanen, Antti Karlsson, Kari Pulkki, Lila Kallio, Petri Virolainen, Arto Mannermaa, Sami Heikkinen, Veli-Matti Kosma, Eija Laakkonen, Jari Laukkanen, Teijo Kuopio, Urho Kujala, Eeva Kangasniemi, Johanna Mäkelä, Jarmo Ritari, Jukka Partanen, Kati Hyvärinen, Mikko Arvas, Anne Pitkäranta, Anu Loukola, Eero Punkka, Miika Koskinen, Olli Carpén, Aino Salminen, David Rice, Johanna Mattson, Joni A. Turunen, Juha Sinisalo, Katariina Hannula-Jouppi, Lauri Aaltonen, Marja-Riitta Taskinen, Martti Färkkilä, Paula Kauppi, Pekka Nieminen, Pentti Tienari, Pirkko Pussinen, Sampsa Pikkarainen, Sanna Seitsonen, Terhi Ollila, Tiinamaija Tuomi, Tuula Salo, Ulla Palotie, Juha Rinne, Kaj Metsärinne, Klaus Elenius, Leena Koulu, Markku Voutilainen, Markus Juonala, Sirkku Peltonen, Ulvi Gursoy, Vesa Aaltonen, Johannes Kettunen, Katri Pylkäs, Marita Kalaoja, Miia Turpeinen, Minna Karjalainen, Raisa Serpi, Reetta Hinttala, Riitta Kaarteenaho, Seppo Vainio, Tuomo Mantere, Anne Remes, Juhani Junttila, Kaisa Tasanen, Kirsi Sipilä, Laura Huilaja, Marja Luodonpää, Nina Hautala, Peeter Karihtala, Saila Kauppila, Terttu Harju, Timo Blomster, Vuokko Anttonen, Hilkka Soininen, Ilkka Harvima, Jussi Pihlajamäki, Kai Kaarniranta, Liisa Suominen, Margit Pelkonen, Maria Siponen, Markku Laakso, Mikko Hiltunen, Mikko Kiviniemi, Päivi Auvinen, Päivi Mäntylä, Reetta Kälviäinen, Valtteri Julkunen, Annika Auranen, Airi Jussila, Hannele Uusitalo-Järvinen, Hannu Kankaanranta, Hannu Uusitalo, Jukka Peltola, Mika Kähönen, Tarja Laitinen, Teea Salmi, Elina Järvensivu, Essi Kaiharju, Hannele Mattsson, Kati Kristiansson, Lotta Männikkö, Markku Laukkanen, Markus Perola, Päivi Laiho, Regis Wong, Sini Lähteenmäki, Sirpa Soini, Teemu Niiranen, Teemu Paajanen, Terhi Kilpi, Tero Hiekkalinna, Tuuli Sistonen, Veikko Salomaa, Masahiro Kanai, Wei Zhou, Tomi Mäkelä, Manuel Rivas, Kimmo Palin, Harri Siirtola, Javier Garcia-Tabuenca, Csilla Sipeky, Dhanaprakash Jambulingam, Johanna Schleutker, Samuel Heron, Venkat Subramaniam Rathinakannan, Aarno Palotie, Jaakko Kaprio, Heikki Joensuu, Mark Daly, Samuli Ripatti

**Affiliations:** 1 Institute for Molecular Medicine Finland, FIMM, HiLIFE, University of Helsinki, Helsinki, Finland; 2 Breast Surgery Unit, Comprehensive Cancer Center, Helsinki University Hospital, Helsinki, Finland; 3 University of Helsinki, Helsinki, Finland; 4 Helsinki Institute for Information Technology HIIT and Department of Mathematics and Statistics, University of Helsinki, Helsinki, Finland; 5 Department of Public Health, University of Helsinki, Helsinki, Finland; 6 Estonian Genome Center, Institute of Genomics, University of Tartu, Tartu, Estonia; 7 Psychiatric & Neurodevelopmental Genetics Unit, Department of Psychiatry, Analytic and Translational Genetics Unit, Department of Medicine, and the Department of Neurology, Massachusetts General Hospital, Boston, MA USA; 8 Broad Institute of MIT and Harvard, Cambridge, MA USA; 9 Comprehensive Cancer Center, Helsinki University Hospital, Helsinki, Finland; 10 Abbvie, North Waukegan Road, North Chicago, Chicago, IL 60064 USA; 11 Astra Zeneca, Francis Crick Avenue, Cambridge Biomedical Campus, Cambridge, CB2 0AA UK; 12 Biogen, 225 Binney Street Cambridge, Cambridge, MA 02142 USA; 13 Bristol-Meyers-Squibb, 430 East 29th Street, New York, NY 10016 USA; 14 Celgene, 86 Morris Avenue, Summit, NJ 07901 USA; 15 Genentech, 1 DNA Way, South San Francisco, CA 94080 USA; 16 GlaxoSmithKline, 980 Great West Road, Brentford, Middlesex TW8 9GS UK; 17 Janssen Biotech, 800/850 Ridgeview Drive, Horsham, PA 19044 USA; 18 Maze Therapeutics, 171 Oyster Point Blvd Suite 300, South San Francisco, CA 94080 USA; 19 Merck, 2000 Galloping Hill Road, Kenilworth, NJ 07033 USA; 20 Pfizer, 235 East 42nd Street, New York, NY 10017 USA; 21 Sanofi, The Finnish Institute for Health and Welfare, Paris, France; 22 Business Finland, Porkkalankatu 1, 00180 Helsinki, Finland; 23 Auria Biobank, University of Turku, Hospital District of Southwest Finland, Yliopistonmäki, FI-20014 Turku, Finland; 24 Biobank of Eastern Finland, University of Eastern Finland, Northern Savo Hospital District, P.O. Box 100, FI-70029 KYS Kuopio, Finland; 25 Central Finland Biobank, University of Jyväskylä, Central Finland Health Care District, Keskussairaalantie 19, FI-40620 Jyväskylä, Finland; 26 Finnish Clinical Biobank Tampere, University of Tampere, Pirkanmaa Hospital District, P.O. Box 2000, FI-33521 Tampere, Finland; 27 Finnish Red Cross Blood Service, Finnish Hematology Registry and Clinical Biobank, Kivihaantie 7, FI-00310 Helsinki, Finland; 28 Helsinki Biobank, Helsinki University and Hospital District of Helsinki and Uusimaa, P.O. Box 400, FI-00029 HUS Helsinki, Finland; 29 Hospital District of Helsinki and Uusimaa, P.O. Box 400, FI-00029 HUS Helsinki, Finland; 30 Hospital District of Southwest Finland, P.O. Box 52, FI-20521 Turku, Finland; 31 Northern Finland Biobank Borealis, University of Oulu, Northern Ostrobothnia Hospital District, P.O. Box 50, FI-90029 Oulu, Finland; 32 Northern Ostrobothnia Hospital District, P.O. Box 10, FI-90029 OYS Oulu, Finland; 33 Northern Savo Hospital District, P.O. Box 100, FI-70029 KYS Kuopio, Finland; 34 Pirkanmaa Hospital District, P.O. Box 2000, FI-33521 Tampere, Finland; 35 THL Biobank/The Finnish Institute for Health and Welfare, P.O. Box 30, 00271 Helsinki, Finland; 36 HiLIFE, University of Helsinki, P.O.Box 3, FI-00014 Helsinki, Finland; 37 University of Stanford, 1265 Welch Road MC5464, MSOB West Wing, Third Floor, Stanford, CA 94305-5464 USA; 38 University of Tampere, P.O. Box 2000, FI-33521 Tampere, Finland; 39 University of Turku, Yliopistonmäki, FI-20014 Turku, Finland

**Keywords:** Cancer genetics, Breast cancer

## Abstract

Polygenic risk scores (PRS) for breast cancer have potential to improve risk prediction, but there is limited information on their utility in various clinical situations. Here we show that among 122,978 women in the FinnGen study with 8401 breast cancer cases, the PRS modifies the breast cancer risk of two high-impact frameshift risk variants. Similarly, we show that after the breast cancer diagnosis, individuals with elevated PRS have an elevated risk of developing contralateral breast cancer, and that the PRS can considerably improve risk assessment among their female first-degree relatives. In more detail, women with the c.1592delT variant in *PALB2* (242-fold enrichment in Finland, 336 carriers) and an average PRS (10–90^th^ percentile) have a lifetime risk of breast cancer at 55% (95% CI 49–61%), which increases to 84% (71–97%) with a high PRS ( > 90^th^ percentile), and decreases to 49% (30–68%) with a low PRS ( < 10^th^ percentile). Similarly, for c.1100delC in *CHEK2* (3.7–fold enrichment; 1648 carriers), the respective lifetime risks are 29% (27–32%), 59% (52–66%), and 9% (5–14%). The PRS also refines the risk assessment of women with first-degree relatives diagnosed with breast cancer, particularly among women with positive family history of early-onset breast cancer. Here we demonstrate the opportunities for a comprehensive way of assessing genetic risk in the general population, in breast cancer patients, and in unaffected family members.

## Abstract (web summary)

Identifying women at high risk of breast cancer has important implications for screening. Here, the authors demonstrate that polygenic risk scores improve breast cancer risk prediction in the population, in women with mutations in high-risk genes and in women with close relatives with the disease.

## Introduction

In women, breast cancer is the most commonly diagnosed cancer and the leading cause of cancer-related deaths^1^. Approximately 5–10% of all breast cancers are estimated to develop due to high-impact germline mutations in breast cancer susceptibility genes, with up to 30% due to pathogenic mutations in *BRCA1* and *BRCA2* and with a smaller proportion carrying mutations in other susceptibility genes, such as *PTEN, TP53*, *CHEK2, PALB2* and *STK11*^2^. While pathogenic mutations in *BRCA1* and *BRCA2* are less common in Finns^3^, two frameshift mutations, c.1592delT (rs180177102) in *PALB2* and c.1100delC (rs555607708) in *CHEK2* have an unusually high allele frequency in Finland, which provides a unique opportunity to explore the impact of these mutations in the population. *PALB2* (Partner and Localizer of *BRCA2*) encodes a key tumour suppressor protein that functions through affecting *BRCA2* nuclear localisation and DNA damage response functions, and through interacting with *BRCA1*^4^. The second gene, *CHEK2* (Checkpoint kinase 2), is a tumour suppressor gene encoding a serine/threonine-protein kinase involved in DNA repair, cell cycle arrest and apoptosis^5^.

Beyond genetic predisposition caused by high-risk mutations in breast cancer susceptibility genes, breast cancer has a highly polygenic mode of inheritance. Large-scale genetic screens have to date identified over a hundred loci associated with risk of breast cancer^6^. These variants, and many more yet to be discovered, represent common genetic variation acting through a wide range of molecular pathways, in contrast to the rare, high-risk pathogenic variants in high-risk breast cancer susceptibility genes that often disrupt a specific pathway involved in maintaining integrity of DNA repair processes. Individually, the common variants have very small effect sizes with odds ratios usually ranging from 0.85 to 1.20, but their cumulative impact in breast cancer risk has been shown to be considerably larger^7^. This cumulative effect can be captured in a single measure by a polygenic risk score (PRS), the summed contribution of many common risk variants, which is able to identify women at over 3-fold risk of breast cancer, compared to women with an average risk^7,8^. By improving identification of these women at high risk of breast cancer, it could serve as a new tool for personalised, risk-based breast cancer screening^7,9,10^.

Here, we comprehensively assess the impact of germline genetic variation on risk of breast cancer and show (1) how a high breast cancer PRS compares to high-risk mutations in breast cancer susceptibility genes, (2) how the PRS modifies the risk of breast cancer in women carrying pathogenic mutations in the *PALB2* and *CHEK2* genes and (3) that the PRS has utility for informing about risk of contralateral breast cancer, and about the risk in first-degree relatives. We use data from the FinnGen study, which combines nationwide health registries with genomic information for 122,978 women from across the country, representing 5% of the Finnish adult female population.

## Results

We studied 122,978 women in FinnGen, with the mean age at the end of follow-up 58.5 (inter-quartile range, IQR 45.1–72.2, range 16.0–106.0). In FinnGen, 8401 (6.8%) women have been diagnosed with breast cancer, with mean age at disease onset of 58.6 (IQR 50.4–66.3, range 21.3–98.3 years). We first tested the association of three polygenic risk scores on breast cancer risk: a 313 SNP score^7^, a genome-wide score by Mars et al.^10^ derived using LDpred software and a new genome-wide score derived using PRS-CS^11^. In our data, the genome-wide scores outperformed the 313 SNP score with hazard ratio (HR) estimates per standard deviation at 1.55 (95% confidence interval, CI 1.52–1.58), 1.63 (CI 1.60–1.67) and 1.71 (CI 1.68–1.75) of the PRS for 313 SNP score, LDpred score and PRS-CS score, each scaled separately to mean zero and unit variance. We therefore chose the PRS built with PRS-CS for subsequent analyses (Table 1 and Supplementary Table 1).

**Table 1:** Comparison of effect sizes for three polygenic risk scores (PRS) with and without excluding the *PALB2* and *CHEK2* loci and regions around them.

	HR (95% CI) *PALB2* and *CHEK2* included	HR (95% CI) PALB2 and CHEK2 excluded
PRS_313_	1.55 (1.52–1.58)	1.54 (1.51–1.57)
PRS_LDpred_	1.63 (1.60–1.67)	1.59 (1.55–1.62)
PRS_CS_	1.71 (1.68–1.75)	1.71 (1.68–1.75)

Out of the three PRSs tested, the PRS built with the software PRS-CS had the strongest association with breast cancer, and was therefore chosen for our analyses. Each PRS was scaled separately to mean zero and unit variance to obtain hazard ratios (HR) per standard deviation. To have a PRS independent of the *PALB2* and *CHEK2* variants, we excluded the variants within the *CHEK2* gene ±3 Mb, and variants within the *PALB2* gene ±2 Mb.

*CI* confidence interval, *PRS*_*313*_ a PRS with 313 genetic variants, with 260 variants polymorphic in FinnGen, *PRS*_*LDpred*_ a PRS built with the software LDpred, *PRS*_*CS*_ a PRS built with the software PRS-CS.

We then studied the allele frequencies, geographic variation and risk estimates for the two Finnish-enriched, high-impact breast cancer mutations. The allele frequency for rs180177102 (*PALB2)* was 0.0014 (242-fold enrichment compared to non-Finnish non-Estonian Europeans, NFEE^12^), with 336 heterozygote mutation carriers included in the analyses. The allele frequency for rs555607708 (*CHEK2*) was 0.0064 (3.7 times enriched in Finns compared to NFEE), with 1641 heterozygotes and 7 homozygote individuals.

### Geographic variation of genetic risk

**Fig. 1:**
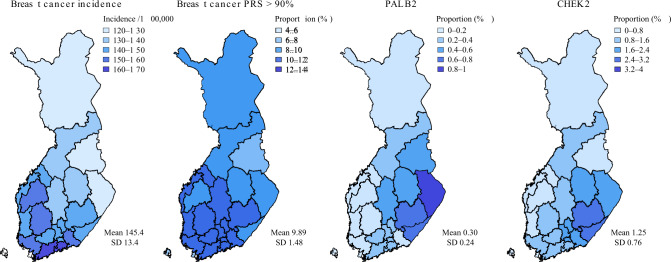
Geographic variation in genetic risk. The risk is compared to age-standardised breast cancer incidence. The proportion of women with the breast cancer polygenic risk score (PRS) above the 90th percentile in each region is estimated with respect to the PRS distribution of the whole country. The *PALB2* and *CHEK2* maps show across different regions the proportion of women carrying at least one risk allele for the variants. The areas represent region of birth obtained from Statistics Finland. The national breast cancer incidence in women was obtained from the Finnish Cancer Registry (publicly available at https://cancerregistry.fi/statistics/) with diagnosis C50 (International Classification of Diseases for Oncology, 3rd edn, ICD-O-3). The incidence represents the mean of 5-year age-standardised incidences (based on the 2014 Finnish population, calculated for each hospital district over 1998–2007). The mean and standard deviation were calculated over the different regions. Variants: rs180177102 (c.1592delT) for *PALB2* and rs555607708 (c.1100delC) for *CHEK2*. *CHEK2* and polygenic risk score plots are based on 122,978 women, and *PALB2* on 109,371 women. Colour contrasts were chosen approximately based on the standard deviation for each map.

Considering Finns have passed internal genetic bottlenecks, we first aimed to characterise any geographic distribution for both the *PALB2* and *CHEK2* mutations, and for the PRS. Both the *PALB2* and *CHEK2* mutations had more carriers in Eastern Finland, with the proportion of carriers ranging from close to 0 in Western Finland, to 2.8% for *CHEK2* in South Karelia and to 0.8% for *PALB2* in North Karelia (Fig. 1). In contrast, we observed slightly higher proportions of individuals with high PRS in Western and Southern Finland, in line with breast cancer incidence.

### The effect of frameshift mutations in PALB2 and CHEK2

Both *PALB2* and *CHEK2* conferred considerably elevated risk for breast cancer (Table 2). The *PALB2* variant conferred a risk increase for breast cancer with a HR of 4.99 (95% CI 4.02–6.20, *p* = 6.76 × 10^−48^), corresponding to a lifetime risk by age 80 of 56.1% (95% CI 50.8–61.4%). The *CHEK2* variant conferred a risk increase for breast cancer with HR 2.19 (95% CI 1.91–2.51), *p* = 3.90 × 10^−29^), corresponding to a lifetime risk of 31.7% (95% CI 29.5–33.9%). Comparing to women with a PRS between the 10th and 90th percentiles (lifetime risk 15.5%, 95% CI 15.3–15.7%), women with PRS above the 90th percentile had a similar effect size as *CHEK2* mutation carriers (HR 2.38, 95% CI 2.26–2.50, *p* = 1.98 × 10^−230^) and their similar lifetime risk was similar (32.5%, 95% CI 31.6–33.4%). However, a high PRS affected a nearly 7-fold larger group of women (Table 1; results excluding first-degree relatives in Supplementary Table 2). Estimating these while accounting for competing risks (non-breast cancer related death) yielded 4.6%, 4.9% and 3.2% lower estimates for lifetime risks in carriers of the *PALB2* and *CHEK2* mutations, and women with high PRS, respectively (Supplementary Figs. 1–3 and Supplementary Table 3).

**Table 2:** Risk for breast cancer events in the population in carriers of the *PALB2* and *CHEK2* frameshift mutations, and in the top decile of the polygenic risk score (PRS).

	*PALB2*	*CHEK2*	PRS > 90%
Number of individuals	336	1648	12,298
Number of cases	84	214	1821
Lifetime risk of breast cancer, % (95% CI)	56.1 (50.8–61.4)	31.7 (29.5–33.9)	32.5 (31.6–33.4)
Mean age at disease onset in cases (SD)	53.1 (10.4)	56.5 (12.0)	57.8 (11.3)

Lifetime risk was estimated by age 80. The variants were rs180177102 (c.1592delT) for *PALB2* and rs555607708 (c.1100delC) for *CHEK2*. The *PALB2* analysis was done in 109,371 women, and the *CHEK2* and PRS analyses in 122,978 women.

*CI* confidence interval, *SD* standard deviation.

### PRS modifies the risk in PALB2 and CHEK2 mutation carriers

**Fig. 2:**
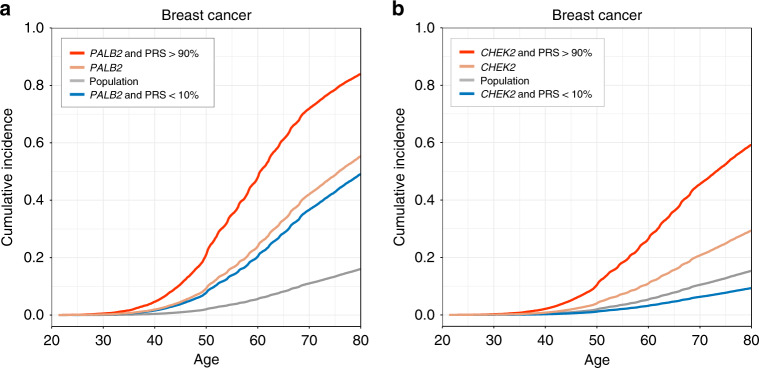
The impact of polygenic risk in *PALB2* and *CHEK2* mutation carriers. Adjusted survival curves showing how the polygenic risk score (PRS) affects the breast cancer risk conferred by the *PALB2* (panel A) and *CHEK2* (panel B) frameshift mutations. Population level was defined as women with PRS between the 10th and 90th percentiles. The *PALB2* analysis was done in 109,371 women and *CHEK2* analysis in 122,978 women. Adjusted survival curves Cox proportional hazards model.

Next, we estimated how the PRS modifies breast cancer risk in the mutation carriers. For both *PALB2* and *CHEK2*, a high PRS further increased the breast cancer risk. In terms of lifetime risk for breast cancer by age 80, women with the *PALB2* mutation and average PRS (10–90th percentile) had a lifetime risk of 55.3% (95% CI 49.4–61.2%), which increased to 83.9% (71.2–96.6%) among women with a high PRS (>90th percentile), and decreased to 49.1% (30.6–67.6%) in women with a low PRS (<10th percentile; Fig. 2 and Tables 3 and 4). Women with *CHEK2* and an average PRS had a lifetime risk of 29.3% (95% CI 26.8–31.8%) which doubled to 59.2% (52.1–66.3%) in women with a high PRS and decreased to 9.3% (4.5–14.1%) in women with low PRS.

**Table 3:** Impact of polygenic risk score (PRS) on the breast cancer risk conferred by the *PALB2* frameshift mutation.

	HR (95% CI)	*p* value	Lifetime risk, % (95% CI)	Cases	Controls
*PALB2* & PRS >90%	11.8 (6.95–20.0)	5.14 × 10^−20^	83.9 (71.2–96.6)	14	18
*PALB2*	4.82 (3.77–6.16)	5.66 × 10^−36^	55.3 (49.4–61.2)	65	211
PRS 10–90%	1.00 (reference)	–	16.0 (15.8–16.2)	5605	81,645
*PALB2* & PRS <10%	4.00 (1.66–9.63)	0.002	49.1 (30.6–67.6)	5	23

Population level was defined as women with PRS between the 10th and 90th percentiles. The estimates were obtained from a Cox proportional hazards model on 109,371 women, without adjusting for multiple comparisons. All tests were two-tailed.

**Table 4:** Impact of polygenic risk score (PRS) on the breast cancer risk conferred by the *CHEK2* frameshift mutation.

	HR (95% CI)	*p* value	Lifetime risk, % (95% CI)	Cases	Controls
*CHEK2* & PRS >90%	5.71 (4.33–7.52)	5.31 × 10^−35^	59.2 (52.1–66.3)	51	133
*CHEK2*	2.12 (1.81–2.48)	2.35 × 10^−20^	29.3 (26.8–31.8)	158	1163
PRS 10–90%	1.00 (reference)	–	15.3 (15.1–15.5)	6116	90,945
*CHEK2* & PRS <10%	0.58 (0.24–1.41)	0.23	9.3 (4.5–14.1)	5	138

Population level was defined as women with PRS between the 10th and 90th percentiles. The estimates were obtained from a Cox proportional hazards model in 122,978 women, without adjusting for multiple comparisons. All tests were two-tailed.

To test for possible interaction between mutation carriers and the PRS, we first compared the PRS effect size in pooled mutation carriers (*PALB2* and *CHEK2)* and in non-carriers. In both carriers and non-carriers, hazard ratios for the top and bottom decile of the PRS were very similar (reference group PRS 10–90%; Table 5). This was observed also in *PALB2* and *CHEK2* mutation carriers separately. For *PALB2*, the HR per SD in carriers was 1.81 (95% CI 1.34–2.44, *p* = 1.05 × 10^−4^), in carriers of *CHEK2*, 1.86 (1.60–2.16, *p* = 6.58 × 10^−16^), and in carriers of neither the *PALB2* nor the *CHEK2* mutation, the HR was 1.71 (1.67–1.74, *p* < 1.00 × 10^−300^). Similarly, in a formal test for interaction by introducing an interaction term in the regression model, we found no evidence of an interaction between the PRS and mutations for neither the *PALB2* variant (*p* = 0.18), nor the *CHEK2* variant (*p* = 0.45).

**Table 5:** To test for interaction in all 122,978 women, we compared the polygenic risk score (PRS) effect size in pooled mutation carriers (pooling *PALB2* and *CHEK2*) and in non-carriers.

	PRS < 10%	PRS 10–90%	PRS > 90%
Mutation	0.42 (0.23–0.79)	1.00 (reference)	2.44 (1.82–3.28)
No mutation	0.38 (0.34–0.43)	1.00 (reference)	2.37 (2.25–2.50)

The table shows the hazard ratios and 95% confidence intervals for the bottom and top deciles, comparing them to women with an average risk (PRS between the 10th and 90th percentiles).

### PRS refines risk assessment in first-degree relatives

**Fig. 3:**
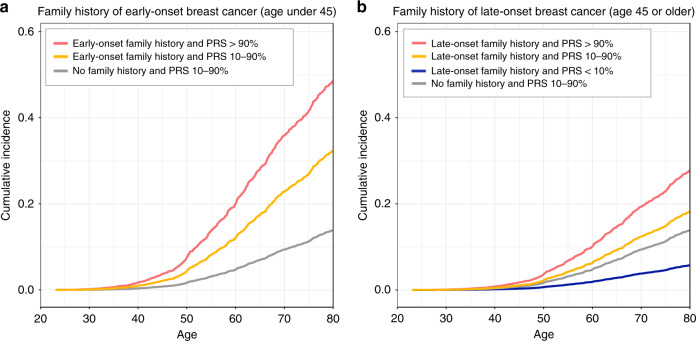
Impact of the polygenic risk score (PRS) in estimating the breast cancer risk of women with a first-degree relative diagnosed with breast cancer. a Shows the impact of family history of early-onset breast cancer, and **b** the impact of family history of late-onset breast cancer. Adjusted survival curves based on Cox proportional hazards models. Risk estimated in 7715 mother–daughter pairs and 12,086 full sibling-pairs (sisters). The pairs of first-degree relatives were inferred with KING by a kinship coefficient ranging between 0.177 and 0.354 (inference based on 57 K unlinked variants). Due to the sample size, we were unable to assess impact of a low PRS (<10th percentile) with early-onset family history.

Next, we evaluated how the PRS modifies the risk conferred by a positive first-degree family history. Family history was assessed in 7715 mother–daughter pairs and 12,086 pairs of sisters, separately for family history of early-onset (age < 45) and late-onset (age ≥ 45) breast cancer. For both, PRS stratified women for breast cancer risk, but the stratification was more pronounced in family history of early-onset disease (Fig. 3 and Supplementary Table 4). Women with an average PRS (between the 10th and 90th percentiles) and positive family history of early-onset breast cancer had a lifetime risk at 32.5% (95% CI 24.0–41.0%) – a risk similar to women with a high PRS (>90th percentile) in the full dataset (32.5%, 31.6–33.4%). A combination of family history of early-onset breast cancer and a high PRS further increased the risk to 49.0% (30.1–67.9%), but with only one breast cancer case in the bottom decile we were unable to estimate the impact of a low PRS. We then tested whether family history adds to risk assessment if we know the woman’s PRS. When adjusting with a continuous PRS, the effect size for family history of early-onset breast cancer was attenuated, from HR 2.80 (95% CI 1.81–4.33, *p* = 4.08 × 10^−6^), to HR 2.32 (1.50–3.60, *p* = 1.72 × 10^−4^). Also for late-onset, the association was attenuated, from HR 1.30 (1.07–1.57, *p* = 0.01), to HR 1.09 (0.90–1.33, *p* = 0.37).

### High PRS increases risk for contralateral breast cancer

Lastly, we tested the association between the PRS contralateral breast cancer among breast cancer patients. With PRS between the 10th and 90th percentile as reference, a high PRS (>90th percentile) was associated with risk of contralateral breast cancer with HR 1.60 (95% CI 1.25–2.04, *p* = 0.0002), with 97 individuals out of 1604 cases with a high PRS being diagnosed with contralateral breast cancer.

## Discussion

Using large-scale biobank data combining longitudinal nationwide health registry data with genomic information, we show that over the life course, the breast cancer PRS strongly alters the breast cancer incidence in high-impact mutation carriers. After breast cancer diagnosis, individuals with an elevated PRS have an increased likelihood of developing contralateral breast cancer, and the PRS can considerably improve risk assessment among their female first-degree relatives.

The breast cancer PRS strongly altered the risk of breast cancer in *PALB2* and *CHEK2* mutation carriers, substantially increasing the risk of breast cancer in women with a high PRS, and lowering the risk in women with a low PRS. Deciding on appropriate surveillance and risk-reduction strategies is a clinical challenge particularly for moderate-risk mutations such as those in *CHEK2*^13^, and our results show that additional information provided by the PRS could guide in these decisions. A combination of breast cancer PRS in the top decile and a mutation in the *CHEK2* variant increased the lifetime risk to 59% – a risk comparable to that seen in *PALB2* mutation carriers – whereas those with a PRS in the bottom decile had a risk similar to the population level.

That PRS modifies the risk in *PALB2* and *CHEK2* mutation carriers supports previous findings suggesting that common genetic variation at least partly explains the widely observed incomplete penetrance of mutations in breast cancer susceptibility genes^14–17^. This variation is now measurable on an individual level with the breast cancer PRS, which captures a wide range of molecular pathways. Our results are in line with previous studies on *BRCA1*, *BRCA2*, *PALB2* and *CHEK2* mutation carriers, but these studies have used a case–control setting or PRSs consisting of <100 variants^16–19^. We conducted the study in a large longitudinal dataset with 120,000 women, using a more predictive, genome-wide PRS and leveraging the considerable enrichment of the *PALB2* and *CHEK2* variants in an isolated population. With the longitudinal setting, we were also able quantify the lifetime risk in *PALB2* and *CHEK2* mutation carriers based on observed events over the life course, instead of calculating them using baseline risks from published studies^17,18^.

Harbouring pathogenic mutations in high-risk breast cancer susceptibility genes often prompt intensified medical surveillance and consideration of preventative procedures such as risk-reducing surgery. The lifetime risk estimates for individuals in the top decile of the PRS was comparable to *CHEK2* mutation carriers – both had a risk of 32% by age 80. Considering this, our results also argue for the need of studies on the impact of targeted actions in women with a high PRS only, who currently go undetected. After the diagnosis, patients with elevated PRS had a 1.6-fold elevated risk for contralateral cancer, providing additional evidence of increased breast cancer susceptibility, a finding that might warrant intensified or prolonged surveillance in breast cancer cases with elevated PRS. This finding is in line with earlier studies showing that familial factors contribute to the risk of contralateral breast cancer^20–22^.

The proportion of mutation carriers and the elevated PRS showed differing geographical distributions. While the elevated PRS distribution followed the breast cancer incidence distribution with highest rates in the early-settlement region in South-Western Finland, the allele frequencies for the *PALB2* and *CHEK2* mutations were highest in the late-settlement region in Eastern Finland. It is likely that the *PALB2* and *CHEK2* mutations have survived both the founder bottleneck in Finland, and the internal bottleneck in the Eastern Finland, therefore being heavily enriched in the Finnish population. These regional differences in both PRS and mutation frequency distributions may have an impact on regional screening strategies.

Finally, the PRS improved risk assessment of first-degree relatives of women with breast cancer, with pronounced stratification particularly for family history of early-onset disease. Family history is an essential factor guiding screening strategies of family members of breast cancer patients^23^, and our results show that PRS could improve the precision of this assessment.

Our study has several limitations. Our findings are limited to individuals of European ancestry and it is important to study the applicability of the results in individuals of admixed and non-European ancestry^24^. The FinnGen study is a mixture of population-based cohorts and samples from hospital biobanks. It is possible that the sampling may introduce biases in some of the estimates. We observed a slightly higher baseline risk compared to the NORDCAN database^25^. However, our key PRS estimates were similar when estimated in a FinnGen subset of population-based cohorts only. Moreover, accounting for the competing risk of mortality from other causes yielded slightly lower estimates for lifetime risks.

In conclusion, we show that a high breast cancer PRS comes with a comparable risk profile to frameshift mutations in breast cancer susceptibility genes *PALB2* and *CHEK2*, and that the PRS strongly modifies breast cancer risk in the mutation carriers. Even after the breast cancer diagnosis, the PRS was associated with breast cancer susceptibility by increasing the risk of contralateral breast cancer, and it considerably improved risk assessment among the patient’s first-degree relatives. These results demonstrate opportunities for a more comprehensive way of assessing genetic risk in the general population, in breast cancer patients and in unaffected family members of breast cancer patients. Optimisation of these strategies in the clinical setting warrant further study.

## Methods

### Participants and endpoints

The data comprised of 122,978 Finnish women in the FinnGen, Data Freeze 5. FinnGen comprises prospective epidemiological cohorts (initiated as far back as 1992), disease-based cohorts, and hospital biobank samples (Supplementary Table 5). The unique national personal identification number links the genotypes to the Finnish Cancer Registry (available from 1953, with nationwide completeness of solid tumours at 96%^26^), as well as to the national hospital discharge registry (1968-), the national death registry (1969-) and the medication reimbursement registry (1964-). These registries cover the whole population.

Breast cancer cases were identified through the Finnish Cancer Registry with diagnosis C50 (International Classification of Diseases for Oncology, 3rd Edition; ICD-O-3), from the drug reimbursement registry by selecting individuals with a reimbursement code for breast cancer, and from the death registry with ICD-10 C50. Contralateral breast cancer was defined as breast cancer in the opposite breast diagnosed over 6 months after the date of the primary breast cancer diagnosis, obtained from the Cancer Registry.

### Genotyping and imputation

FinnGen samples were genotyped with Illumina and Affymetrix arrays (Illumina Inc., San Diego, and Thermo Fisher Scientific, Santa Clara, CA, USA), and genotype calls were made with the GenCall or zCall (for Illumina) and the AxiomGT1 algorithm for Affymetrix data. Individuals with ambiguous gender, high genotype missingness (>5%), excess heterozygosity (+-4SD) and non-Finnish ancestry were excluded, as well as all variants with high missingness (>2%), low Hardy–Weinberg equilibrium *p*-value (<1e-6) and minor allele count (MAC < 3). Array data pre-phasing was carried out with Eagle 2.3.5^27^ with the number of conditioning haplotypes set to 20,000. Genotype imputation was done with Beagle 4.1^28^ (as described in 10.17504/protocols.io.xbgfijw) by using the SISu v3 population-specific reference panel developed from high-quality data for 3,775 high-coverage (25-30x) whole-genome sequences in Finns.

### Variants

We chose two previously reported Finnish-enriched frameshift variants for our main analyses, rs180177102 (c.1592delT) in *PALB2* and rs555607708 (c.1100delC) in *CHEK2*. Genotype data batches with an imputation INFO score <0.8 were excluded. This excluded 13,607 women from analyses involving the *PALB2* variant (mainly older disease-based cohorts), but no exclusions were needed for *CHEK2*. *PALB2* mutation carrier status was ignored in analyses involving the *CHEK2* variant, and vice versa. Women homozygous for the *CHEK2* variant were analysed jointly with the heterozygotes.

### Polygenic risk score

To choose our breast cancer PRS, we compared three scores: (1) a previously published PRS with 313 SNPs^7^, (2) another previously published, genome-wide PRS^10^ built with the software LDpred^29^ and (3) a genome-wide PRS we built with the software PRS-CS (PRS-CS-auto, with 1000 Genomes Project European sample, *N* = 503, as the external LD reference panel) using HapMap3 variants^11^. For the LDpred and PRS-CS PRSs, the input weights came from a large independent genome-wide association study (GWAS)^6^. To have a PRS independent of the *PALB2* and *CHEK2* variants, we excluded the variants within the *CHEK2* gene ±3 Mb, and variants within the *PALB2* gene ±2 Mb (Supplementary Fig. 4). Out of these three, the PRS-CS score showed the strongest association for breast cancer and was therefore chosen for subsequent analyses (Table 1 and Supplementary Table 1). All three PRSs showed acceptable goodness-of-fit (Supplementary Fig. 5). The final variant count for the PRS-CS PRS with *PALB2* and *CHEK2* excluded was 1,074,667.

A high PRS was defined as a PRS above the 90th percentile, as it corresponds to a lifetime risk of ≥30%, which guidelines consider as the threshold for high risk^23^. Correspondingly, we defined a PRS below the 10th percentile as a low PRS. Individuals between the 10th to 90th percentiles served as the reference category.

### Geographic variation

Geographic variation is reported by region of birth (obtained from Statistics Finland) as the proportion of individuals with (1) the frameshift mutations in the *PALB2* or *CHEK2* variants, and (2) high PRS (>90th percentile). The benchmark for these analyses was age-standardised (age in 2014) breast cancer incidence for the whole Finnish population, calculated as the mean of 5-year incidences for each hospital district over 1998–2007. The incidence data was obtained from the Finnish Cancer Registry (publicly available at https://cancerregistry.fi/statistics/). Polygon data for the Finnish map were obtained from GADM (https://gadm.org/data.html).

### Population structure-related bias analysis

A population structure-related bias analysis was performed by following the approach described in detail in Kerminen et al.^30^. In brief, the method measures the accumulation of PRS differences between the Western and Eastern subpopulations of Finland using a “random PRS”, made from a randomly chosen set of independent (*r*^2^ < 0.1) variants with minor allele frequency >0.05 that are not associated with breast cancer (breast cancer GWAS^6^ *p*-value >0.5). If such random PRS accumulated differences between the subpopulations, that could indicate a population genetic bias in effect estimates of the GWAS, rather than a real difference in genetic susceptibility of breast cancer between the subpopulations. We found no evidence of such bias (Supplementary Fig. 6), which indicates that any detected geographic variation in the PRS is unlikely to result from a population genetic bias.

### Risk assessment in first-degree relatives

The pairs of first-degree relatives were inferred with KING v2.2.4^31^ by a kinship coefficient ranging between 0.177 and 0.354 (inference based on 57 K unlinked variants). To analyse the impact of family history in first-degree relatives, we randomly sampled one female relative for each woman who had at least one first-degree relative in the dataset. For mother–daughter pairs, the mother was assigned as the index relative. For sisters, we randomly assigned one to be the index relative, irrespective of age. If both women in the pair were breast cancer cases, we used the year of diagnosis to assign the woman diagnosed earlier as the index. Some individuals appeared several times as non-index individuals, which may occur when, for instance, a woman is the daughter of one index individual and the sister of another – we therefore randomly sampled the data to contain each non-index individual only once. We then inferred the risk of breast cancer in these unique non-index individuals. We analysed separately family history of early-onset (age < 45) and late-onset (age ≥ 45) breast cancer.

### Statistical analysis

We estimated HRs and 95% CIs with the Cox proportional hazards model, and used Schoenfeld residuals and log–log inspection for assessing the proportional hazards assumption. Start of follow-up was set at birth, and follow-up ended at the first record of the endpoint of interest, death or at the end of follow-up on 31 December 2018, whichever came first. All tests were two-tailed. In all survival analyses, we used age as the time scale, with 63 batches and the first 10 principal components as covariates. The only exception was the analysis on contralateral breast cancer, where follow-up started from the diagnosis, and age was included as a covariate.

Goodness-of-fit for the PRS was assessed with a method proposed by May & Hosmer for a Cox proportional hazards model^32^. In line with previous studies on breast cancer susceptibility genes, we assessed lifetime risk (cumulative incidence without competing risks) by age 80^14,33^. Lifetime risk was estimated from the adjusted survival curves, with 95% CIs obtained by normal approximation. The adjusted survival curves were plotted with the R package *survminer*. This presents the expected survival curves separately for subgroups, based on the Cox model. To estimate the covariate-adjusted cumulative incidence functions in the presence of competing risks, we used the Stata module *stcompadj*^34^. The competing event was non-breast cancer causes of death and covariates were assumed to have similar effects the main and competing event.

Interactions between the PRS and the pathogenic mutations were assessed (1) by comparing the PRS effect sizes in pooled and non-pooled mutation carriers and non-carriers (with the PRS scaled to zero mean and unit variance within the whole dataset), and (2) formally by introducing an interaction term for the mutation and the continuous PRS. For data and variant handling and PRS calculation, we used BCFtools versions 1.7 and 1.9, and PLINK 2.0. For statistical analyses, we used R 3.6.3 and Stata 16.0 (College Station, TX, USA). Cromwell and WOMtool were used for workflow handling.

### Ethics statement

The FinnGen project is approved by the Finnish Institute for Health and Welfare (THL), approval number THL/2031/6.02.00/2017, amendments THL/1101/5.05.00/2017, THL/341/6.02.00/2018, THL/2222/6.02.00/2018, THL/283/6.02.00/2019), Digital and population data service agency VRK43431/2017-3, VRK/6909/2018-3, the Social Insurance Institution (KELA) KELA 58/522/2017, KELA 131/522/2018, KELA 70/522/2019 and Statistics Finland TK-53-1041-17.

Patients and control subjects in FinnGen provided informed consent for biobank research, based on the Finnish Biobank Act. Alternatively, older research cohorts, collected prior the start of FinnGen (in August 2017), were collected based on study-specific consents and later transferred to the Finnish biobanks after approval by Valvira, the National Supervisory Authority for Welfare and Health. Recruitment protocols followed the biobank protocols approved by Valvira. The Ethics Review Board of the Hospital District of Helsinki and Uusimaa approved the FinnGen study protocol Nr HUS/990/2017.

The Biobank Access Decisions for FinnGen samples and data utilised in FinnGen Data Freeze 5 include: THL Biobank BB2017_55, BB2017_111, BB2018_19, BB_2018_34, BB_2018_67, BB2018_71, BB2019_7 Finnish Red Cross Blood Service Biobank 7.12.2017, Helsinki Biobank HUS/359/2017, Auria Biobank AB17-5154, Biobank Borealis of Northern Finland_2017_1013, Biobank of Eastern Finland 1186/2018, Finnish Clinical Biobank Tampere MH0004, Central Finland Biobank 1-2017 and Terveystalo Biobank STB 2018001. Analyses of potential geographic bias of PRS were done with THL biobank permission BB2019_44.

### Reporting summary

Further information on research design is available in the Nature Research Reporting Summary linked to this article.

## Supplementary information

Supplementary Information

Reporting Summary

## Data Availability

The FinnGen data may be accessed through Finnish Biobanks’ FinBB portal (web link: www.finbb.fi, email: info.fingenious@finbb.fi). The GWAS summary statistics used for constructing our main PRS are available at http://bcac.ccge.medschl.cam.ac.uk/bcacdata/oncoarray/oncoarray-and-combined-summary-result/gwas-summary-results-breast-cancer-risk-2017/, with contact information at http://bcac.ccge.medschl.cam.ac.uk/contact/. The weights for our main PRS are available at PGS Catalog (pgs-info@ebi.ac.uk) at https://www.PGSCatalog.org/score/PGS000335/, and the previously published PRSs at https://www.PGSCatalog.org/score/PGS000004/ and https://www.PGSCatalog.org/score/PGS000332/. The remaining data are available within the Article, Supplementary Information or available from the authors upon request.

## Code availability

The full genotyping and imputation protocol for FinnGen is described at 10.17504/protocols.io.xbgfijw.

## References

[1] Bray F (2018). Global cancer statistics 2018: GLOBOCAN estimates of incidence and mortality worldwide for 36 cancers in 185 countries. CA Cancer J. Clin..

[2] Economopoulou P, Dimitriadis G, Psyrri A (2015). Beyond BRCA: new hereditary breast cancer susceptibility genes. Cancer Treat. Rev..

[3] Vehmanen P (1997). Low proportion of *BRCA1* and *BRCA2* mutations in finnish breast cancer families: evidence for additional susceptibility genes. Hum. Mol. Genet..

[4] Ducy M (2019). The tumor suppressor *PALB2*: Inside out. Trends Biochem. Sci..

[5] Nevanlinna H, Bartek J (2006). The *CHEK2* gene and inherited breast cancer susceptibility. Oncogene.

[6] Michailidou K (2017). Association analysis identifies 65 new breast cancer risk loci. Nature.

[7] Mavaddat N (2019). Polygenic risk scores for prediction of breast cancer and breast cancer subtypes. Am. J. Hum. Genet..

[8] Khera AV (2018). Genome-wide polygenic scores for common diseases identify individuals with risk equivalent to monogenic mutations. Nat. Genet..

[9] Lee A (2019). BOADICEA: A comprehensive breast cancer risk prediction model incorporating genetic and nongenetic risk factors. Genet. Med..

[10] Mars N (2020). Polygenic and clinical risk scores and their impact on age at onset and prediction of cardiometabolic diseases and common cancers. Nat. Med..

[11] Ge T, Chen CY, Ni Y, Feng YA, Smoller JW (2019). Polygenic prediction via Bayesian regression and continuous shrinkage priors. Nat. Commun..

[12] Karczewski KJ (2020). The mutational constraint spectrum quantified from variation in 141,456 humans. Nature.

[13] Tung N (2016). Counselling framework for moderate-penetrance cancer-susceptibility mutations. Nat. Rev. Clin. Oncol..

[14] Antoniou AC (2014). Breast-cancer risk in families with mutations in *PALB2*. New Engl. J. Med..

[15] Antoniou AC (2002). A comprehensive model for familial breast cancer incorporating *BRCA1*, *BRCA2* and other genes. Br. J. Cancer.

[16] Kuchenbaecker, K. B. et al. Evaluation of polygenic risk scores for breast and ovarian cancer risk prediction in *BRCA1* and *BRCA2* mutation carriers. *J. Natl. Cancer Inst*. **109**, djw302 (2017). 10.1093/jnci/djw302 PMC5408990 28376175

[17] Muranen TA (2017). Genetic modifiers of *CHEK2**1100delC-associated breast cancer risk. Genet. Med..

[18] Gallagher S (2020). Association of a polygenic risk score with breast cancer among women carriers of high- and moderate-risk breast cancer genes. JAMA Netw. Open.

[19] Fahed AC (2020). Polygenic background modifies penetrance of monogenic variants for tier 1 genomic conditions. Nat. Commun..

[20] Reiner AS (2018). Breast cancer family history and contralateral breast cancer risk in young women: An update from the women’s environmental cancer and radiation epidemiology study. J. Clin. Oncol..

[21] Narod SA, Kharazmi E, Fallah M, Sundquist K, Hemminki K (2016). The risk of contralateral breast cancer in daughters of women with and without breast cancer. Clin. Genet..

[22] Robson, M. E. et al. Association of common genetic variants with contralateral breast cancer risk in the WECARE study. *J. Natl. Cancer Inst.* 109, djx051 (2017). 10.1093/jnci/djx051 PMC5939625 28521362

[23] National Collaborating Centre for Cancer. NICE clinical guidelines, no. 164. Familial breast cancer: classification and care of people at risk of familial breast cancer and management of breast cancer and related risks in people with a family history of breast cancer (2013). 25340237

[24] Martin AR (2019). Clinical use of current polygenic risk scores may exacerbate health disparities. Nat. Genet..

[25] Danckert, B. et al. NORDCAN: cancer incidence, mortality, prevalence and survival in the Nordic countries, version 8.2 (26.03.2019). http://www.dep.Iarc.Fr/nordcan/ Accessed on 13 July 2020.

[26] Leinonen MK, Miettinen J, Heikkinen S, Pitkaniemi J, Malila N (2017). Quality measures of the population-based Finnish cancer registry indicate sound data quality for solid malignant tumours. Eur. J. Cancer.

[27] Loh PR (2016). Reference-based phasing using the Haplotype Reference Consortium panel. Nat. Genet..

[28] Browning BL, Browning SR (2016). Genotype imputation with millions of reference samples. Am. J. Hum. Genet..

[29] Vilhjalmsson BJ (2015). Modeling linkage disequilibrium increases accuracy of polygenic risk scores. Am. J. Hum. Genet..

[30] Kerminen S (2019). Geographic variation and bias in the polygenic scores of complex diseases and traits in Finland. Am. J. Hum. Genet..

[31] Manichaikul A (2010). Robust relationship inference in genome-wide association studies. Bioinformatics.

[32] May S, Hosmer DW (1998). A simplified method of calculating an overall goodness-of-fit test for the Cox proportional hazards model. Lifetime Data Anal..

[33] Yang, X. et al. Ovarian and breast cancer risks associated with pathogenic variants in RAD51C and *RAD51D. J. Natl. Cancer Inst*. **112**, djaa030 (2020). 10.1093/jnci/djaa030 PMC7735771 32107557

[34] Coviello E. stcompadj: Stata Module To Estimate The Covariate-adjusted Cumulative Incidence Function In The Presence Of Competing Risks. Statistical Software Components S457063 (Department of Economics, Boston College, 2009).

