# Supplementary material for: The role of polygenic risk and susceptibility genes in breast cancer over the course of life
Source: Nat Commun. 2020 Dec 14;11:6383. doi: 10.1038/s41467-020-19966-5 (PMC7736877; doi:10.1038/s41467-020-19966-5)
Supplement: Supplementary file 1 — Supplementary Information [file 41467_2020_19966_MOESM1_ESM.pdf]

Supplementary Table 1. Comparison of effect sizes for three polygenic risk scores (PRS) in four different settings. Each PRS was scaled separately to mean zero and unit variance to obtain odds ratios (OR) and hazard ratios (HR) per standard deviation.

	**Number of variants**	**OR (95%CI)*** *PALB2* **and** *CHEK2* **included**	**OR (95%CI)*** *PALB2* **and** *CHEK2* **excluded**	**HR (95%CI)**** **Incident cases in population-based cohorts,** *PALB2* **and** *CHEK2* **excluded**
PRS_{313}	260 / 313	1.61 (1.57-1.64)	1.59 (1.56-1.63)	1.55 (1.45-1.66)
PRS_{LDpred}	6,390,808	1.71 (1.67-1.75)	1.66 (1.62-1.69)	1.67 (1.56-1.79)
PRS_{CS}	1,074,667	1.80 (1.76-1.84)	1.79 (1.75-1.84)	1.79 (1.67-1.92)

CI = confidence interval. PRS_{313} = a PRS with 313 genetic variants, with 260 variants polymorphic in FinnGen when *PALB2* and *CHEK2* loci are included. PRS_{LDpred} = a PRS built with the software LDpred. PRS_{CS} = a PRS built with the software PRS-CS. To have a PRS independent of the *PALB2* and *CHEK2* variants, we excluded the variants within the *CHEK2* gene ±3Mb, and variants within the *PALB2* gene ±2Mb. All models were adjusted with batches and the first ten principal components.

* Adjusted with age at the end of follow-up.

** Total n = 19,280, with 353 prevalent cases excluded, and 816 incident cases included in the analysis. Follow-up started at study enrollment. The mean age at baseline was 49.3 (inter-quartile range, IQR 37.8-60.0), and the mean duration of follow-up was 16.0 (IQR 11.8-21.8). The population-based datasets: THL BIOBANK FINRISK 1992, THL BIOBANK FINRISK 2002, THL BIOBANK FINRISK 1997, THL BIOBANK FINRISK 2007, THL BIOBANK FINRISK 2012, THL BIOBANK HEALTH 2000. For more details on the dataset, see Supplementary Table 5.

Supplementary Table 2. Effect sizes without any exclusions based on relatedness (column ‘No exclusions’; values from Table 2), and after excluding first-degree relatives (column ‘1st degree excluded’). The reference group for columns with the polygenic risk score (PRS) is individuals with a PRS between the 10th and 90th percentiles.

	PALB2		***CHEK2***		**PRS >90%**	
	**No exclusions**	**1st degree excluded**	**No exclusions**	**1st degree excluded**	**No exclusions**	**1st degree excluded**
Number of individuals	336	315	1,648	1,448	12,298	10,681
Number of cases	84	79	214	187	1,821	1,605
Lifetime risk of breast cancer, % (95% CI)	56.1 (50.8-61.4)	56.4 (51.1-61.7)	31.7 (29.5-33.9)	32.0 (29.6-34.4)	32.5 (31.6-33.4)	33.1 (32.2-34)
Hazard ratio (95% CI)	4.99 (4.02-6.20)	4.93 (3.91-6.22)	2.19 (1.91-2.51)	2.16 (1.87-2.51)	2.38 (2.26-2.50)	2.38 (2.25-2.52)
Mean age at disease onset in cases (SD)	53.1 (10.4)	52.6 (10.7)	56.5 (12.0)	56.5 (12.1)	57.8 (11.3)	57.6 (11.2)

CI = confidence interval, HR = hazard ratio, SD = standard deviation. After excluding men from the genotype file, the 1st degree relatedness was inferred with KING (based on 57K variants , parameter options --unrelated --degree 1). After this exclusion, 106,803 of the 122,978 women remained in the dataset (95,802 in the *PALB2* dataset; see Methods for details on *PALB2* data exclusions based on imputation INFO scores). The PRS was scaled to zero mean and unit variance separately within the 106,803 women. Variants: rs180177102 (c.1592delT) for *PALB2* and rs555607708 (c.1100delC) for *CHEK2*.

Supplementary Table 3. Lifetime risk with 95% confidence intervals for breast cancer events in carriers of the *PALB2* and *CHEK2* frameshift mutations, and in the top decile of the polygenic risk score (PRS). The lifetime risks are presented separately with and without accounting for competing risks (non-breast cancer related death). The estimates accounting for competing risks correspond to the survival curves in Supplementary Figures 1 to 3.

	PALB2	***CHEK2***	**PRS >90%**
Lifetime risk of breast cancer	56.1 (50.8-61.4)	31.7 (29.5-33.9)	32.5 (31.6-33.4)
Lifetime risk of breast cancer accounting for competing risks	51.5 (46.1-56.8)	26.8 (24.6-28.9)	29.3 (28.5-30.1)

Lifetime risk estimated by age 80. Variants: rs180177102 (c.1592delT) for *PALB2* and rs555607708 (c.1100delC) for *CHEK2*. *PALB2* analysis was done in 109,371 women and *CHEK2* analysis in 122,978 women.

Supplementary Table 4. Impact of polygenic risk score (PRS) in estimating the breast cancer risk of women with a first-degree relative diagnosed with breast cancer. The definition for family history of early-onset breast cancer was age <45 at diagnosis, and for family history of late-onset breast cancer, age ≥45.

	HR (95% CI)	p	Lifetime risk, % (95% CI)	Cases	Controls
Early-onset family history and PRS >90%	4.70 (2.08-10.6)	1.96×10^{-4}	49.0 (30.1-67.9)	6	21
Early-onset family history and PRS 10-90%	2.69 (1.57-4.58)	2.89×10^{-4}	32.5 (24.0-41.0)	14	103
No family history and PRS 10-90%	1.00 (reference)	-	14.0 (13.4-14.6)	659	14,062
Early-onset family history and PRS <10%	-	-	-	1	6
Late-onset family history and PRS >90%	2.21 (1.52-3.22)	3.28×10^{-5}	27.8 (21.7-33.9)	29	179
Late-onset family history and PRS 10-90%	1.35 (1.08-1.69)	0.01	18.3 (15.9-20.7)	85	937
No family history and PRS 10-90%	1.00 (reference)	-	14.0 (13.4-14.6)	659	14,062
Late-onset family history and PRS <10%	0.39 (0.01-1.69)	0.18	5.8 (0.4-11.2)	2	70

Due to the sample size, we were unable to assess impact of a low PRS (<10th percentile) with early-onset family history. Adjusted survival curves based on Cox proportional hazards models. Risk estimated in in 7,715 mother-daughter pairs and 12,086 full sibling-pairs (sisters). The pairs of first-degree relatives were inferred with KING by a kinship coefficient ranging between 0.177 and 0.354 (inference based on 57K unlinked variants).

Supplementary Table 5. Cohorts and biobanks within FinnGen Data Freeze 5.

**Cohort / biobank**	**N**
AURIA BIOBANK*	13,413
BIOBANK OF CENTRAL FINLAND*	891
BIOBANK OF EASTERN FINLAND*	3,651
BLOOD SERVICE BIOBANK	17,187
BOREALIS BIOBANK*	3,066
HELSINKI BIOBANK*	27,564
OTHER	2
TAMPERE BIOBANK*	4,395
TERVEYSTALO BIOBANK	69
THL BIOBANK BOTNIA	3,530
THL BIOBANK COROGENE	1,717
THL BIOBANK FINHEALTH 2017	3,107
THL BIOBANK FinIPF	61
THL BIOBANK FINRISK 1992	2,683
THL BIOBANK FINRISK 1997	3,585
THL BIOBANK FINRISK 2002	3,817
THL BIOBANK FINRISK 2007	2,781
THL BIOBANK FINRISK 2012	2,760
THL BIOBANK GENERISK	4,454
THL BIOBANK HEALTH 2000	3,654
THL BIOBANK HEALTH 2011	405
THL BIOBANK KUUSAMO	77
THL BIOBANK MIGRAINE	4,946
THL BIOBANK SUPER	4,243
THL BIOBANK T1D	4,900
THL BIOBANK TWINS	6,020
**Sum**	**122,978**

*Hospital-based biobanks

Supplementary Figure 1. The covariate-adjusted cumulative incidence in the presence of competing risks (non-breast cancer causes of death) for *PALB2*.

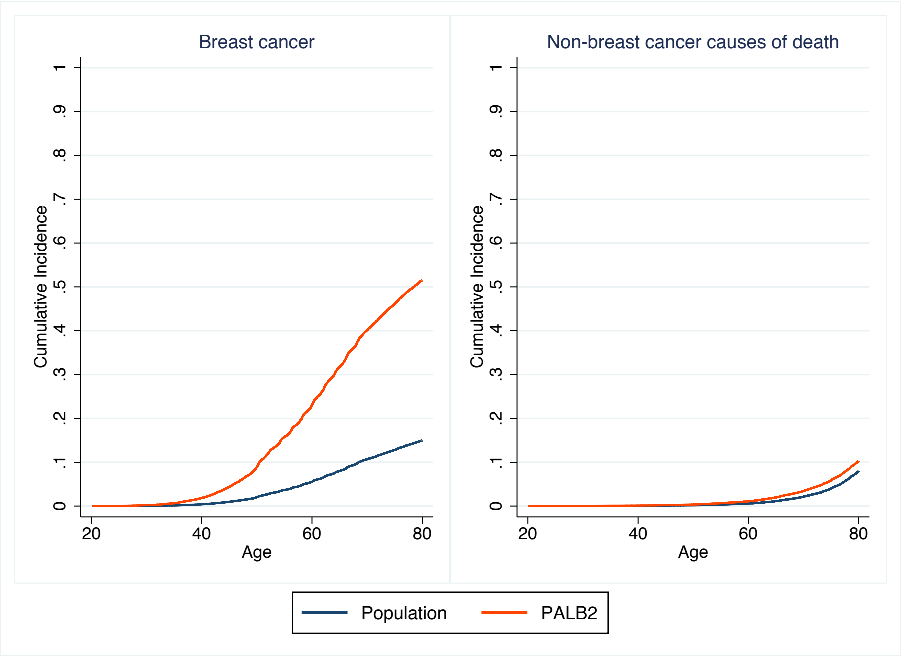

Population level defined as women without the *PALB2* mutation. In accordance with Table 2, the analysis was done in 109,371 women, with 84 of the 336 *PALB2* mutation carriers being diagnosed with breast cancer over the follow-up. The competing event was observed in 13 *PALB2* mutation carriers and in 3,217 individuals within the population group. To estimate the covariate-adjusted cumulative incidence functions in the presence of competing risks, we used the Stata module stcompadj.[1]

Supplementary Figure 2. The covariate-adjusted cumulative incidence in the presence of competing risks (non-breast cancer causes of death) for *CHEK2*.

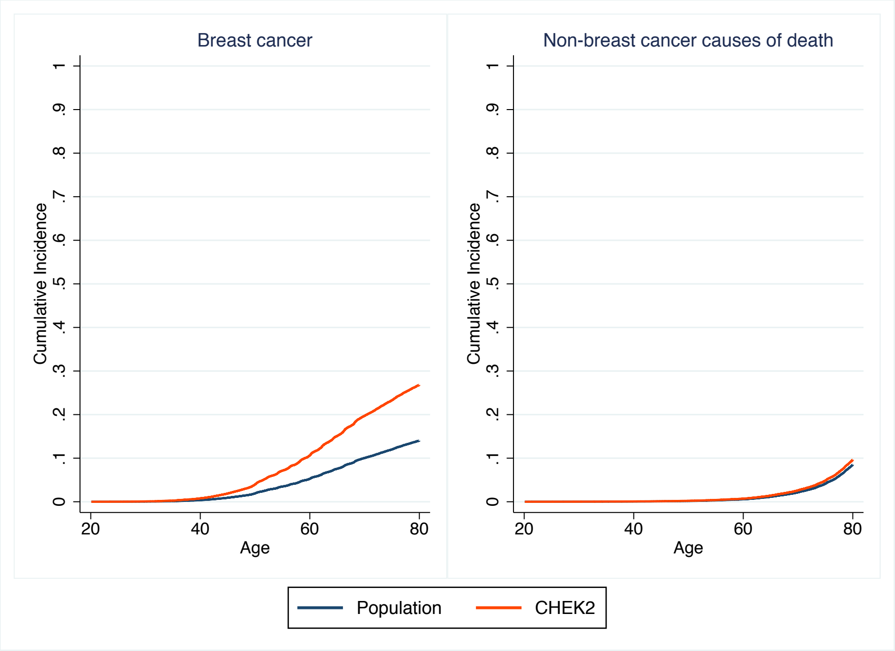

Population level defined as women without the *CHEK2* mutation. In accordance with Table 2, the analysis was done in 122,978 women, with 214 of the 1,648 *CHEK2* mutation carriers being diagnosed with breast cancer over the follow-up. The competing event was observed in 78 *CHEK2* mutation carriers and in 5,085 individuals within the population group. To estimate the covariate-adjusted cumulative incidence functions in the presence of competing risks, we used the Stata module stcompadj.[1]

Supplementary Figure 3. The covariate-adjusted cumulative incidence in the presence of competing risks (non-breast cancer causes of death) for high polygenic risk score (PRS), defined as a PRS above the 90th percentile.

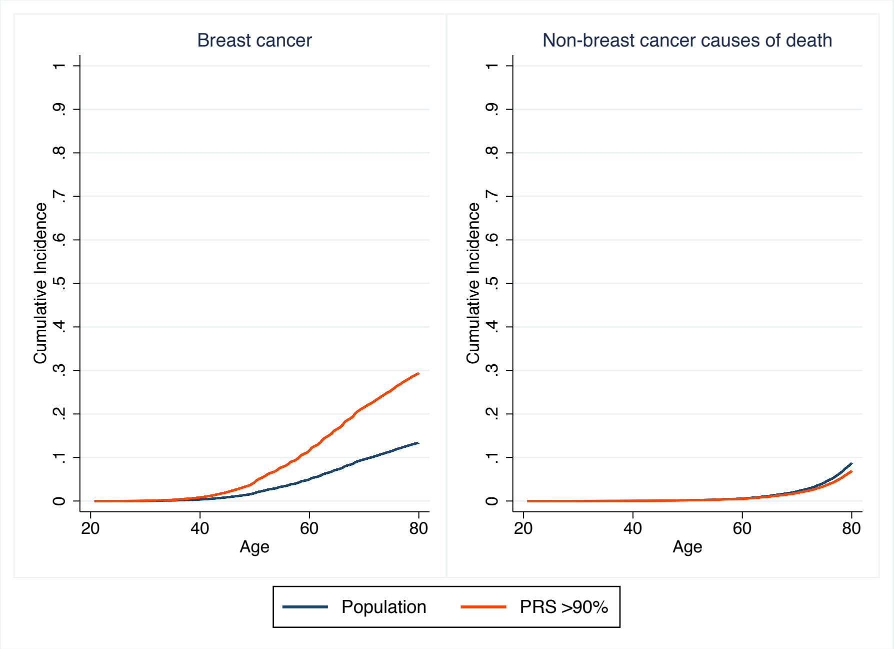

Population level defined as women with PRS between the 10th and 90th percentiles. In accordance with Table 2, the analysis was done in 122,978 women, with 1,821 of the 12,298 women with a high PRS being diagnosed with breast cancer over the follow-up. The competing event was observed in 454 women with a high PRS and in 4,143 individuals within the population group. To estimate the covariate-adjusted cumulative incidence functions in the presence of competing risks, we used the Stata module stcompadj.[1]

Supplementary Figure 4. The impact of excluding the *PALB2* (top row) and *CHEK2* (bottom row) loci from the polygenic risk score (PRS). The purple curve shows the PRS distribution in individuals carrying the mutations, and the gray curve the individuals without the mutation.

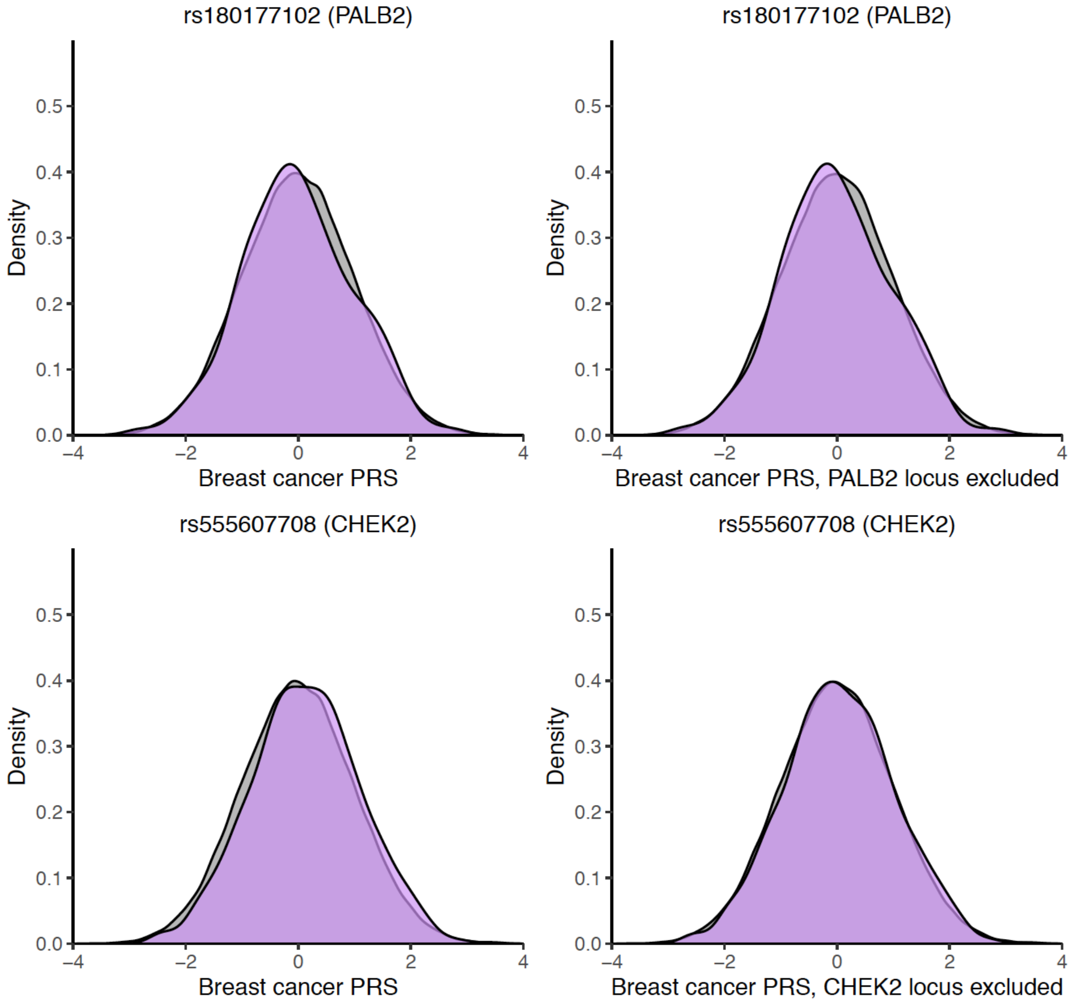

To create a PRS independent of the *PALB2* and *CHEK2* variants, we excluded the *CHEK2* gene ±3Mb, and the *PALB2* gene ±2Mb. For the *PALB2* variant, the data comprised 336 heterozygote individuals, and for *CHEK2*, with 1,641 heterozygotes and 7 homozygote individuals. In line with the rest of the analyses, women homozygous for the *CHEK2* variant were analysed jointly with the heterozygotes.

Supplementary Figure 5. Goodness-of-fit of the breast cancer polygenic risk score (PRS) for any breast cancer, breast cancer mortality, and non-localized breast cancer at diagnosis. The x-axis and y-axis represent the expected and observed proportions of cases, respectively. The PRS is divided into 20 bins of equal size.

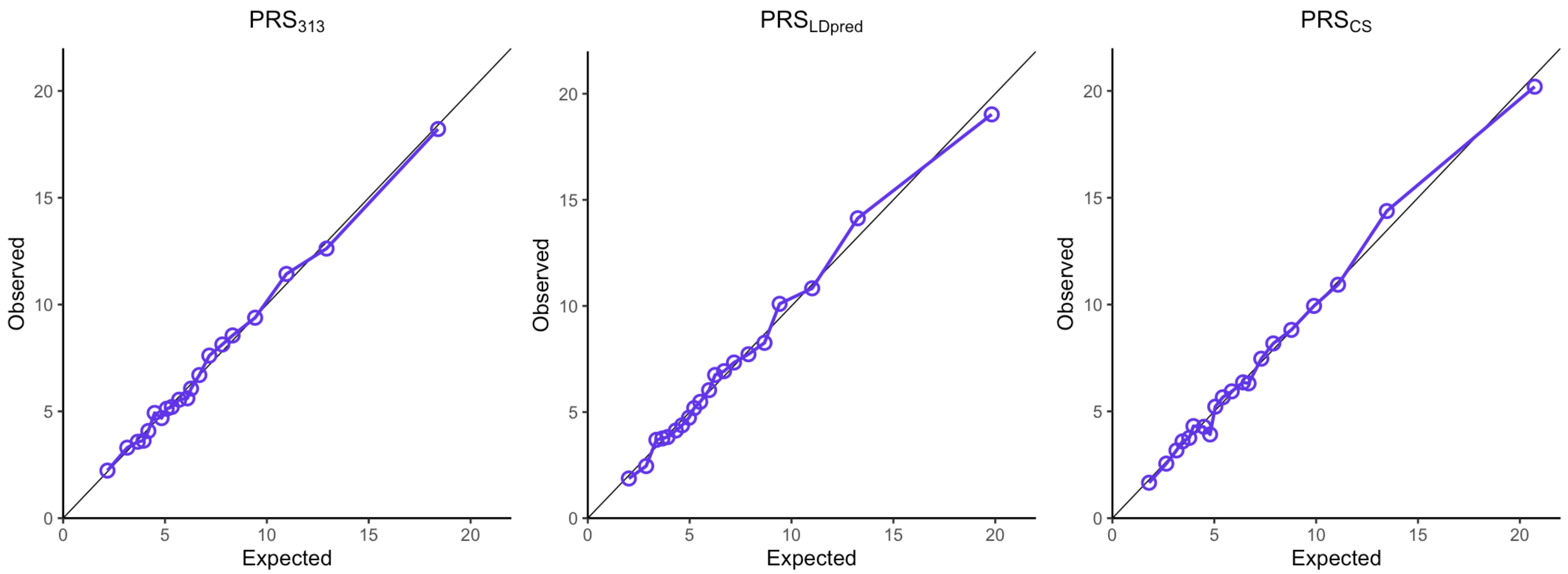

PRS**313** = a PRS with 313 genetic variants, with 260 variants available in FinnGen. PRS**LDpred** = a PRS built with the software LDpred. PRS**CS** = a PRS built with the software PRS-CS. *PALB2* and *CHEK2* loci not excluded. Goodness-of-fit for the Cox proportional hazards model was assessed with the R package *survMisc* using the function *gof()*, which calculates the expected incidence in each of the 20 bins, based on a coxph() object. In the regression analysis, we used a continuous PRS to estimate effects per standard deviation, and used age as the time scale, with batches and the first ten principal components of ancestry as covariates.

Supplementary Figure 6. Population structure-related bias analysis. The absolute value between Western and Eastern populations using different numbers of independent variants (r^2 <0.1) randomly chosen with p-value >0.5 and minor allele frequency >0.05, using the summary statistics for the genome-wide association study on breast cancer by Michailidou et al.[6] The solid region is the 95% probability interval under the theoretical null assumption of zero effect sizes and completely independent variants (r^2 = 0), defined by calculating the average of empirically estimated null region in Kerminen et al.[2] Points show the mean and error bars the range over 10 random scores.

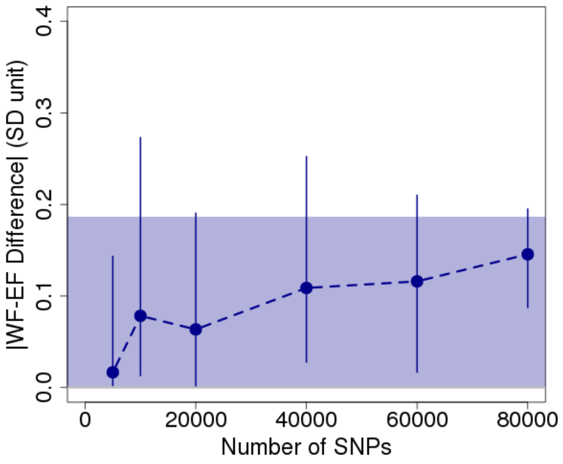

WF = Western Finland, EF = Eastern Finland, SNP = single nucleotide polymorphism. In line with the main breast cancer PRS, the summary statistics used for generating the random scores exclude the *CHEK2* gene ±3Mb, and the *PALB2* gene ±2Mb. The points on the x axis correspond to 5,000, 10,000, 20,000, 40,000, 60,000, and 80,000 numbers of SNPs.

Supplementary references

1. Coviello E. Stcompadj: STATA module to estimate the covariate-adjusted cumulative incidence function in the presence of competing risks. *Statistical Software Components S457063, Department of Economics, Boston College*. 2009
2. Kerminen S, Martin AR, Koskela J, Ruotsalainen SE, Havulinna AS, Surakka I, et al. Geographic variation and bias in the polygenic scores of complex diseases and traits in Finland. *Am. J. Hum. Genet.* 2019;104:1169-1181
