# Supplementary material for: The role of polygenic risk and susceptibility genes in breast cancer over the course of life
Source: Nat Commun. 2020 Dec 14;11:6383. doi: 10.1038/s41467-020-19966-5 (PMC7736877; doi:10.1038/s41467-020-19966-5)
Supplement: Supplementary file 2 — Reporting Summary [file 41467_2020_19966_MOESM2_ESM.pdf]

nature research

Corresponding author(s): Ripatti, S

Last updated by author(s): Oct 15, 2020

Reporting Summary

Nature Research wishes to improve the reproducibility of the work that we publish. This form provides structure for consistency and transparency in reporting. For further information on Nature Research policies, see our Editorial Policies and the Editorial Policy Checklist.

Statistics

For all statistical analyses, confirm that the following items are present in the figure legend, table legend, main text, or Methods section.

n/a	Confirmed	
☐	☒	The exact sample size (*n*) for each experimental group/condition, given as a discrete number and unit of measurement
☐	☒	A statement on whether measurements were taken from distinct samples or whether the same sample was measured repeatedly
☐	☒	The statistical test(s) used AND whether they are one- or two-sided<br>*Only common tests should be described solely by name; describe more complex techniques in the Methods section.*
☐	☒	A description of all covariates tested
☐	☒	A description of any assumptions or corrections, such as tests of normality and adjustment for multiple comparisons
☐	☒	A full description of the statistical parameters including central tendency (e.g. means) or other basic estimates (e.g. regression coefficient) AND variation (e.g. standard deviation) or associated estimates of uncertainty (e.g. confidence intervals)
☐	☒	For null hypothesis testing, the test statistic (e.g. F, t, r) with confidence intervals, effect sizes, degrees of freedom and P value noted<br>*Give P values as exact values whenever suitable.*
☒	☐	For Bayesian analysis, information on the choice of priors and Markov chain Monte Carlo settings
☒	☐	For hierarchical and complex designs, identification of the appropriate level for tests and full reporting of outcomes
☐	☒	Estimates of effect sizes (e.g. Cohen's *d*, Pearson's *r*), indicating how they were calculated

Our web collection on statistics for biologists contains articles on many of the points above.

Software and code

Policy information about availability of computer code

Data collection	No software was used for data collection.
Data analysis	We used following softwares for imputation, handling the genetic and phenotypic data:<br>Cromwell and WOMtool<br>Plink 2.0<br>BCFtools 1.7 and 1.9<br>Eagle 2.3.5<br>Beagle 4.1 (08Jun17.d8b)<br>R 3.4.1 and 3.6.3<br>Stata 16.0<br>LDpred 0.9.09<br>KING 2.4.4<br>PRS-CS<br>The full genotyping and imputation protocol for FinnGen is described at dx.doi.org/10.17504/protocols.io.nmndc5e<br>For data transformations, visualization and plotting of the results, we used R 3.6.3, including packages ggplot2 and survminer.

For manuscripts utilizing custom algorithms or software that are central to the research but not yet described in published literature, software must be made available to editors and reviewers. We strongly encourage code deposition in a community repository (e.g. GitHub). See the Nature Research guidelines for submitting code & software for further information.

Data

Policy information about availability of data

All manuscripts must include a data availability statement. This statement should provide the following information, where applicable:

- Accession codes, unique identifiers, or web links for publicly available datasets
- A list of figures that have associated raw data
- A description of any restrictions on data availability

The FinnGen data may be accessed through Finnish Biobanks' FinBB portal (web link: www.finbb.fi, email: info.fingenious@finbb.fi). The GWAS summary statistics used for constructing our main PRS are available at http://bcac.ccge.medschl.cam.ac.uk/bcacdata/oncoarray/oncoarray-and-combined-summary-result/gwas-summary-results-breast-cancer-risk-2017/, with contact information at http://bcac.ccge.medschl.cam.ac.uk/contact/. The weights for our main PRS are available at PGS Catalog (pgs-info@ebi.ac.uk) at https://www.PGSCatalog.org/score/PGS000335/, and the previously published PRSs at https://www.PGSCatalog.org/score/PGS000004/ and https://www.PGSCatalog.org/score/PGS000332/. The remaining data are available within the Article, Supplementary Information or available from the authors upon request.

Field-specific reporting

Please select the one below that is the best fit for your research. If you are not sure, read the appropriate sections before making your selection.

[x] Life sciences [ ] Behavioural & social sciences [ ] Ecological, evolutionary & environmental sciences

For a reference copy of the document with all sections, see nature.com/documents/nr-reporting-summary-flat.pdf

Life sciences study design

All studies must disclose on these points even when the disclosure is negative.

Sample size	The study is based on FinnGen data (n = 122,978 women) with 8,401 breast cancer cases. We used all available data for the analyses, and studies have shown that even earlier data freezes (https://doi.org/10.1038/s41591-020-0800-0) with a smaller sample size had been sufficient for polygenic risk score analyses on breast cancer.
Data exclusions	Exclusion of samples and variants was based on standard guidelines and quality control procedures. The pre-established exclusion criteria are descibed in detail in the Methods. To ensure variant data of high quality, genotype data batches with an imputation INFO score <0.8 were excluded. This excluded 13,607 women from analyses involving the PALB2 variant (mainly older disease-based cohorts), but no exclusions were needed for CHEK2. To have a PRS independent of the PALB2 and CHEK2 variants, we excluded the variants within the CHEK2 gene ±3Mb, and variants within the PALB2 gene ±2Mb.
Replication	To benchmark our polygenic risk score, we compared it to two previously published breast cancer polygenic risk scores. Both our PRS and the two previously published PRSs were strongly associated with breast cancer, with the hazard ratios reported in Table 1, and detailed sensitivity analyses reported in Supplementary Table 1.
Randomization	The study does not include allocation of participants to different experimental groups.
Blinding	No blinding was relevant for this observational study.

Reporting for specific materials, systems and methods

We require information from authors about some types of materials, experimental systems and methods used in many studies. Here, indicate whether each material, system or method listed is relevant to your study. If you are not sure if a list item applies to your research, read the appropriate section before selecting a response.

Materials & experimental systems

n/a	Involved in the study	
[x]	[ ]	Antibodies
[x]	[ ]	Eukaryotic cell lines
[x]	[ ]	Palaeontology and archaeology
[x]	[ ]	Animals and other organisms
[ ]	[x]	Human research participants
[x]	[ ]	Clinical data
[x]	[ ]	Dual use research of concern

Methods

n/a	Involved in the study	
[x]	[ ]	ChIP-seq
[x]	[ ]	Flow cytometry
[x]	[ ]	MRI-based neuroimaging

Human research participants

Policy information about studies involving human research participants

Population characteristics	This study included participants from different study cohorts, all of European ancestry. The key characteristics of the participants in the cohorts are described in the main text and in the Supplementary Table 5. The mean age at the end of follow-up was 58.5 (inter-quartile range, IQR 45.1-72.2, range 16.0 to 106.0). In FinnGen, 8,401 (6.8%) women have been diagnosed with breast cancer, with mean age at disease onset of 58.6 (IQR 50.4-66.3, range 21.3 to 98.3 years). In the study, we also characterize the geographic variation in genetic risk (main text and Figure 1).
Recruitment	Random sample of subjects from Finnish population-based and clinical biobanks. A proportion of FinnGen was ascertained through hospital biobanks and disease-based collections, but hazard ratios for polygenic risk score categories and breast cancer prevalences have been reported to be similar between FinnGen and population-based cohorts (https://doi.org/10.1038/s41591-020-0800-0). However, it is possible that the inclusion of the clinical biobank collections lead to some overestimation of lifetime risks. Participation was voluntary. It is possible that the sampling may introduce biases in some of the estimates, but our key polygenic risk score estimates were similar when estimated in a FinnGen subset of population-based cohorts only (Supplementary Table 1).
Ethics oversight	Patients and control subjects in FinnGen provided informed consent for biobank research, based on the Finnish Biobank Act. Alternatively, older research cohorts, collected prior the start of FinnGen (in August 2017), were collected based on study-specific consents and later transferred to the Finnish biobanks after approval by Valvira, the National Supervisory Authority for Welfare and Health. Recruitment protocols followed the biobank protocols approved by Valvira. The Ethics Review Board of the Hospital District of Helsinki and Uusimaa approved the FinnGen study protocol Nr HUS/990/2017.<br><br>The FinnGen project is approved by the Finnish Institute for Health and Welfare (THL), approval number THL/2031/6.02.00/2017, amendments THL/1101/5.05.00/2017, THL/341/6.02.00/2018, THL/2222/6.02.00/2018, THL/283/6.02.00/2019), Digital and population data service agency VRK43431/2017-3, VRK/6909/2018-3, the Social Insurance Institution (KELA) KELA 58/522/2017, KELA 131/522/2018, KELA 70/522/2019 and Statistics Finland TK-53-1041-17.<br><br>The Biobank Access Decisions for FinnGen samples and data utilized in FinnGen Data Freeze 4 include: THL Biobank BB2017_55, BB2017_111, BB2018_19, BB_2018_34, BB_2018_67, BB2018_71, BB2019_7 Finnish Red Cross Blood Service Biobank 7.12.2017, Helsinki Biobank HUS/359/2017, Auria Biobank AB17-5154, Biobank Borealis of Northern Finland_2017_1013, Biobank of Eastern Finland 1186/2018, Finnish Clinical Biobank Tampere MH0004, Central Finland Biobank 1-2017, and Terveystalo Biobank STB 2018001. Analyses of potential geographic bias of PRS were done with THL biobank permission BB2019_44.

Note that full information on the approval of the study protocol must also be provided in the manuscript.
